# Optimal surgical timing and radiotherapy dose for trimodality therapy in locally advanced non‐small cell lung cancer

**DOI:** 10.1002/cam4.4123

**Published:** 2021-08-05

**Authors:** James E. Han, Shaakir Hasan, J. Isabelle Choi, Robert H. Press, Charles B. Simone

**Affiliations:** ^1^ Department of Radiation Oncology University of California Los Angeles Los Angeles CA USA; ^2^ Department of Radiation Oncology New York Proton Center New York NY USA

**Keywords:** lung cancer, radiation dose, surgical timing, survival, trimodality therapy

## Abstract

**Purpose/Objectives:**

Data are conflicting on the effects of time interval from neoadjuvant chemoradiation (NCRT) to surgery for locally advanced non‐small‐cell lung cancer (LA‐NSCLC). This study investigated the impact of surgical timing after NCRT and radiation dose on postoperative mortality and overall survival (OS).

**Materials and Methods:**

Using the National Cancer Database, we identified 3489 LA‐NSCLC patients treated with NCRT and surgery. Multivariate Cox proportional hazards analysis (MVA) was used to examine the effects of surgery >7 weeks from NCRT completion on OS. Propensity score (PS)‐matched survival analysis for surgery ≤7 and >7 weeks was performed. Postoperative mortality was assessed.

**Results:**

Median OS for surgery ≤7 weeks and >7 weeks after NCRT were 56.9 versus 45.6 months (hazard ratio, HR 1.18 [1.07–1.30]; *p* < 0.001). Surgery >7 weeks correlated with decreased OS on MVA (HR 1.15 [1.04–1.27]; *p* = 0.009) and PS matching (HR 1.16 [1.049–1.29]; *p* = 0.004). Time as a continuous variable correlated with OS on MVA (HR 1.003 [1.001–1.006]; *p* = 0.0056) and PS matching (HR 1.004 [1.001–1.006]; *p* = 0.004). Among 2902 lobectomy patients, the mortality rate for surgery ≤66 days was 5.2% versus 8.1% for >66 days (MVA HR 1.59 [1.02–2.49]; *p* = 0.04). Higher neoadjuvant radiotherapy dose correlated with surgery >7 weeks and lobectomy >66 days on MVA.

**Conclusions:**

Increased interval >7 weeks from NCRT to surgery for LA‐NSCLC is correlated with worse OS and lobectomy ≤66 days correlated with improved OS. Surgery ≤7weeks may improve tumor control, whereas higher mortality for surgery >66 days may relate to late NCRT manifestations. Neoadjuvant doses of 44–50.4 Gy may minimize risks of radiation‐induced lung injury and surgical complications and facilitate surgery within the optimal 7‐week interval.

## Graphical abstract

Increased interval >7 weeks between neoadjuvant chemoradiation (NCRT) and surgery for locally advanced non‐small‐cell lung cancer is correlated with worse overall survival (OS), and lobectomy ≤66 days correlated with improved OS. Surgery ≤7 weeks may improve tumor control, whereas higher mortality for surgery >66 days may relate to late NCRT manifestations. Neoadjuvant doses of 44–50.4 Gy may minimize risks of radiation‐induced lung injury and surgical complications and facilitate surgery within the optimal 7‐week interval.

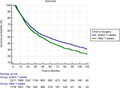

## 1 INTRODUCTION

Several phase II trials^1^, ^2^, ^3^ confirmed the viability and effectiveness of neoadjuvant chemoradiation (NCRT) followed with surgery for trimodality therapy (TMT) in a select population of locally advanced non‐small‐cell lung cancer (LA‐NSCLC) patients. Neoadjuvant therapy can help achieve negative‐margin resections, downstage the type of surgery needed, and even increase the rates of pathological mediastinal downstaging, which correlates with improvements in overall survival (OS).^4^ Although surgery after NCRT can improve progression‐free survival (PFS) rates, this has not translated into an OS benefit to date. In phase III randomized prospective clinical trial, the benefit in PFS reported by Albain et al.^5^ was likely offset by a 26% postoperative mortality rate in pneumonectomy patients, leading to a lack of improved OS in the overall trimodality cohort. It is unclear if the timing of surgery relative to NCRT completion impacted the high perioperative mortality seen in that trial.

In several prospective randomized trials, patients completed surgery 3–5 weeks^1^, ^2^, ^3^, ^5^ after completing NCRT, whereas other studies allowed up to 8 weeks.^6^ Gao et al.^7^ found that delaying surgery more than 6 weeks resulted in decreased OS. In addition, Rice et al.^8^ found that lengthy delays in surgery of >114 days following neoadjuvant therapy resulted in significantly decreased survival. Overall, however, there seems to be a lack of consensus agreement between clinicians regarding the optimal time interval (TI) from NCRT to surgery for patients treated with TMT, likely attributable to the large variations found in these studies and off trial use in individual clinical practice.

In addition, a wide range of NCRT doses exist, and no trials have compared the outcomes of NCRT doses to date. Historically, seminal TMT trials from SWOG^1^, ^2^, ^3^ and Intergroup/RTOG^5^ used radiation doses of 45 Gy. However, Sonett et al.^9^ and Cerfolio et al.^10^ demonstrated safety in utilizing higher doses of ≥59 Gy preoperatively. Furthermore, RTOG 0229,^11^ a single‐arm phase II trial, treated patients to neoadjuvant radiotherapy (RT) doses of 61.2 Gy followed with surgery, resulting in limited toxicity rates and higher than previously reported rates of pathologic mediastinal nodal clearance. Large single‐institution series have suggested definitive doses (≥60 Gy) delivered in the neoadjuvant setting can improve OS.^12^

Despite the paucity of data, TMT with NCRT and surgery remains a widely employed treatment option in select patients with LA‐NSCLC, with no standard optimal TI to surgery or neoadjuvant RT dose established. We examined if TI to surgery following NCRT influences OS and postoperative mortality in LA‐NSCLC patients. In addition, we investigated which factors, including total radiation dose and effect of TI to surgery after NCRT are independent predictors of OS and postoperative mortality.

## 2 MATERIALS AND METHODS

### 2.1 Patient population

The National Cancer Database (NCDB) receives reports of nearly 70% of new cancer cases diagnosed within the United States.^13^, ^14^ We queried all patients with NSCLC diagnosed between the years 2005 and 2014 from the NCDB and abstracted patient data on sex, age, race, comorbidities, socioeconomic status, residential information, treatment facility, diagnosis year, tumor characteristics, and treatment characteristics.

**FIGURE 1:**
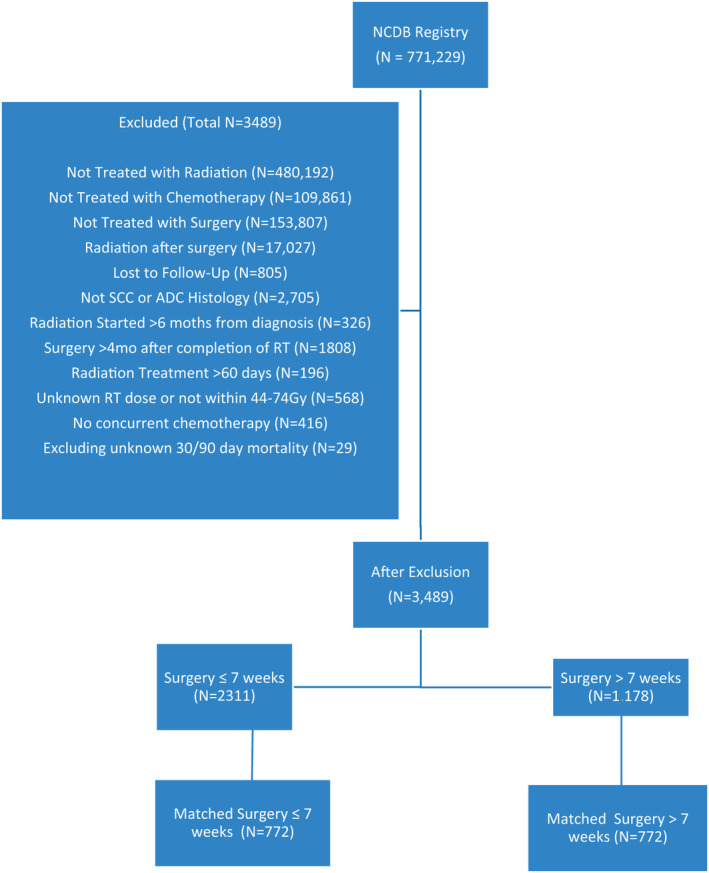
CONSORT diagram of patient cohort. SCC, squamous cell carcinoma, ADC, adenocarcinoma

From the eligible *N* = 771,229 patients having NSCLC, we extracted those with newly diagnosed non‐metastatic NSCLC from 2005 to 2014. Patients not treated with RT, chemotherapy, or surgery were excluded. Patients without dates OF delivery of RT, chemotherapy, and surgery were also excluded as were patients who did not receive NCRT before surgery and whose treatment was not started within 6 months of their diagnoses. RT must have been completed in ≤60 days to a dose between 44 and 77 Gy, and surgery must have been performed ≤4 months after completing NCRT. A treatment duration of 60 days was chosen as this provides a reasonable interval where treatment can be delivered without compromising effectiveness. Patients with non‐invasive tumors as well as all histologies other than squamous cell carcinoma and adenocarcinoma were excluded. Additionally, we excluded patients with no diagnosis, no follow‐up data, or unknown 30/90 day post‐operative mortality. Our final patient cohort included 3489 patients. The cohort composition is shown in Figure 1.

### 2.2 Statistical analysis

We analyzed if TI to surgery after NCRT influences postoperative mortality or OS. We also identified predictors of increased TI to surgery after NCRT and assessed whether neoadjuvant RT dose independently correlates with OS and postoperative mortality in LA‐NSCLC patients. We applied an immortal time bias exclusion of 3 months, which is a standard for population‐based studies. We used a receiver operating characteristic (ROC) analysis to determine the optimal cut point for our analysis and found this to be surgery >7 weeks (49 days) after completing NCRT. Both multivariate Cox proportional hazards analysis (MVA) and logistic regression were used to assess adjusted covariate effects on OS. Also using ROC analysis, we also evaluated postoperative mortality rates for patients specifically treated with lobectomy and found the optimal cut point to be >66 days. Finally, we performed a 1:1 propensity score (PS)‐matched survival analysis for surgery ≤7 weeks and >7 weeks from NCRT.

Analysis of variance and Pearson chi‐square were used to assess continuous and categorical variables by the timing of surgery >7 weeks. Potential predictors of surgery >7 weeks were modeled using logistic regression (univariate and multivariate), including variables such as age, academic center, Charlson–Deyo score (CDS), education, histology, income, nodal disease, sex, race, and primary tumor stage. Categorical values included CDS, location of facility, type of facility, income, population, and race.

### 2.3 Survival analysis

We generated Kaplan–Meier estimates using the time to event curves. Log‐rank test was used to compare outcomes based on demographic and on clinical‐ and treatment‐related variables. Outcome was the OS measured from the date of diagnosis until the date of death or of censor. The median follow‐up time for the cohort was 32.1 months (interquartile range, IQR [6.2–53.1]). Hazard ratio (HR) with Wald 95% confidence interval (CI) was calculated from indicated reference groups. MVA used to examine surgery ≤7 weeks and >7 weeks was adjusted for race, sex, age, co‐morbidities, education, income, insurance type, treatment at academic centers, T‐stage, nodal status, and histology. Furthermore, we performed MVA of variables associated with postoperative mortality rates for patients undergoing lobectomy >66 days. All statistical tests are two‐sided, and a *p* < 0.05 was used to define statistical significance, and Medcalc (version 22) was used to perform our analyses.

### 2.4 Propensity score matching

We characterized two treatment cohorts: patients who underwent surgery ≤7 weeks and >7 weeks after NCRT. We derived the conditional probability of having surgery >7 weeks with a multivariable logistic regression model that included all baseline variables listed previously. We PS‐matched patients 1:1 into surgery ≤7 weeks and >7 weeks groups. Examination of standardized mean differences with mirror histograms was used to assess balance pre‐ and post‐PS matching in baseline covariates.^15^ We evaluated the balances of matched covariates. Standardized differences of <10% were considered sufficiently matched.^16^ Survival evaluation compared surgery >7 weeks using the Kaplan–Meier method using log‐rank test.

## 3 RESULTS

### 3.1 Baseline characteristics, by the timing of surgery

Of the 771,229 patients with LA‐NSCLC collected between 2005 and 2014, 3489 patients were treated with TMT, consisting of NCRT and surgery. The majority were white (88.5%), male (57.1%), with CDS of 0 (64.1%). Among all patients, 34.0% received therapy at an academic center and 45.0% at a comprehensive community cancer center.

From the 3489 total patients, 2311 (66.2%) patients had surgery ≤7 weeks and 1178 (33.8%) >7 weeks after NCRT. Both groups had a median age of 61 years. In general, the surgery ≤7 weeks and >7 weeks groups had similar baseline characteristics (Table 1). However, differences were identified in radiation treatment, with more patients in the surgery >7 weeks group receiving a higher neoadjuvant RT dose. Twenty‐two percent of patients with surgery >7 weeks were treated with a dose between 50.4 and 60 Gy versus 16.1% in surgery ≤7 weeks (*p* < 0.001). Furthermore, 23.8% of patients in the surgery >7 weeks group received a radiation dose >60 Gy versus 9.9% in the surgery ≤7 weeks group (*p* < 0.001).

**TABLE 1:** Baseline patient, tumor, and treatment characteristics

Characteristic	No. (%) *N *= 3489
Clinical factors	
Age at diagnosis, years (median, Interquartile range)	61 (24–88)
Gender	
Male	1992 (57.1)
Female	1497 (42.9)
Charlson–Deyo comorbidity score	
0	2236 (64.1)
1	974 (27.9)
2	240 (6.9)
3	39 (1.1)
Clinical T classification	
T1	463 (13.3)
T2	1175 (33.7)
T3	1157(33.2)
T4	490 (14.0)
Unknown	204 (5.8)
Clinical N classification	
N0	1076 (30.8)
N1	451 (12.9)
N2	1649 (47.3)
N3	85 (2.4)
Unknown	228 (6.5)
Histology	
Squamous cell carcinoma	1757 (50.4)
Adenocarcinoma	1732 (49.6)
Disease laterality	
Left	1408 (40.4)
Right	1994 (57.1)
Unknown	87 (2.5)
Radiation dose (range 44–70)	
≤45 Gy	1206 (34.6)
45–50.4 Gy	1144 (32.8)
50.4–60 Gy	630 (18.1)
>60 Gy	509 (14.6)
Surgery	
Lobectomy	2902 (83.2)
Pneumonectomy	587 (16.8)
Facility type	
Community	275 (8.0)
Comprehensive community	1582(45.0)
Academic	1176 (34.0)
Integrated network	423 (12.0)
Unknown	33 (1.0)
Socioeconomic factors	
Ethnicity	
White	3088 (88.5)
Black	312 (8.9)
Other	89 (2.6)
	
No high school degree	
≥29%	513 (14.7)
≥20%–28.9%	943 (27.0)
≥14%–19.9%	1224 (35.1)
≤14%	761 (21.8)
Unknown	48 (0.01)
Income	
<$30,000	591 (17.0)
$30,000–$34,999	837 (24.0)
$35,000–$45,999	961 (27.5)
$46,000+	1050 (30.1)
Not available	50 (1.4)
Geography	
New England	293 (8.4)
Middle Atlantic	513 (14.7)
South Atlantic	667 (19.1)
East North Central	883 (25.3)
East South Central	304 (8.7)
West North Central	324 (9.3)
West South Central	121 (3.5)
Mountain	113 (3.2)
Pacific	271 (7.8)
Population	
Metro >1,000,000	1638 (46.9)
Urban ≥20,000–250,000	189 (5.4)
Urban not adjacent to metro ≥2500–19,999	108 (3.1)
All others	1554 (44.5)

Note

Numbers might not sum to 100.0% due to rounding.

### 3.2 Correlates of surgery >7 weeks

The correlates of surgery >7 weeks are presented in Table 2. Age (odds ratio [OR] 1.01 [1.00–1.02]; *p* = 0.023), African American ethnicity (OR 1.35 [1.03–1.79]; *p* = 0.035), and CDS of 1 (OR 1.23 [1.03–1.46]; *p* = 0.021) were associated with surgery >7 weeks after NCRT on MVA. Radiation dose in general predicted for surgery >7 weeks. Both doses between 50.4 and 60 Gy (OR 1.90 [1.56–2.32]; *p* < 0.001) and >60 Gy (OR 3.22 [2.60–3.99]; *p* < 0.001) compared to <50.4 Gy had a statistically significant correlation with surgery >7 weeks on MVA.

**TABLE 2:** Predictors of surgery >7 weeks

			Association with AS >7 weeks	
	Treatment group		Binomial regression	
Characteristic	AS ≤ 7 weeks (*N* = 2311)	AS > 7 weeks (*N* = 1178)	HR [95% CI]	*p*
Age (continuous)	61 (24–88)	61 (33–84)	1.01 [1.00–1.02]	**0.029**
Gender				
Male	1319 (57.1)	673 (57.1)	Reference	
Female	992 (42.9)	505 (42.9)	1.04 [0.88–1.22]	0.660
Charlson–Deyo score				
0	1511 (65.4)	725 (61.5)	Reference	
1	621 (26.9)	353 (30.0)	1.23 [1.03–1.46]	**0.021**
2	154 (6.7)	86 (7.3)	1.27 [0.94–1.72]	0.126
	25 (1.1)	14 (1.2)	1.18 [0.59–2.38]	0.642
T stage				
T1	318 (13.8)	145 (12.3)	Reference	
T2	767 (33.2)	408 (34.6)	1.18 [0.92–1.52]	0.186
T3	767 (33.2)	390 (33.1)	1.20 [0.92–1.57]	0.177
T4	312 (13.5)	178 (15.1)	1.23 [0.90–1.67]	0.188
Unknown	147 (6.4)	57 (4.8)	—	—
N stage				
N0	710 (30.7)	366 (31.1)	Reference	
N1	298 (12.9)	153 (13.0)	0.98 [0.76–1.26]	0.890
N2	1098 (47.5)	551 (46.8)	0.91 [0.74–1.12]	0.379
N3	50 (2.2)	35 (3.0)	1.23 [0.75–2.02]	0.410
Unknown	155 (6.7)	73 (6.2)	—	—
Histology				
Adenocarcinoma	1161 (50.2)	571 (48.5)	Reference	
Squamous cell	1150 (49.8)	607 (51.5)	1.09 [0.92–1.28]	
Laterality				
Unknown	58 (2.5)	25 (2.1)	Reference	
Right	1317 (57.0)	677 (57.5)	1.27 [0.73–2.22]	0.397
Left	933 (40.4)	475 (40.3)	1.27 [0.73–2.21]	0.401
Midline	3 (0.1)	1 (0.1)	1.31 [0.13–13.75]	0.819
Surgery				
Lobectomy	1901 (82.3)	1001 (85.0)	Reference	
Pneumonectomy	410 (17.7)	177 (15.0)	0.83 [0.67–1.04]	0.101
Dose (Gy)				
<50.4	1711 (74.0)	639 (54.2)	Reference	
50.4 to 60	371 (16.1)	259 (22.0)	1.90 [1.56–2.32]	**<0.001**
>60	229 (9.9)	280 (23.8)	3.22 [2.60–4.0]	**<0.001**
Ethnicity				
White	2059 (89.1)	1029 (87.4)	Reference	
Black	183 (7.9)	129 (11.0)	1.35 [1.02–1.79]	**0.035**
Other	69 (3.0)	20 (1.7)	0.63 [0.36–1.09]	0.097
Facility type				
Community	177 (7.7)	98 (8.3)	Reference	
Comprehensive	1042 (45.1)	540 (45.8)	0.96 [0.71–1.30]	0.796
Academic	770 (33.3)	406 (34.5)	0.96 [0.70–1.30]	0.773
Integrated	298 (12.9)	125 (10.6)	0.80 [0.56–1.16]	0.237
Unknown	24 (1.0)	9 (0.8)	0.64 [0.24–1.73]	0.380
Income				
<$30,000	357 (15.4)	234 (19.9)	Reference	
$30,000–$34,999	550 (23.8)	287 (24.4)	0.81 [0.62–1.05]	0.111
$35,000–$45,999	642 (27.8)	319 (27.1)	0.83 [0.62–1.11]	0.211
$46,000+	726 (31.4)	324 (27.5)	0.76 [0.54–1.07]	0.116
Unknown	36 (1.6)	14 (1.2)		0.998
No high school diploma				
≥29%	332 (14.4)	181 (15.4)	Reference	
≥20%–28.9%	599 (25.9)	344 (29.2)	1.16 [0.89–1.51]	0.266
≥14%–19.9%	806 (34.9)	418 (35.5)	1.06 [0.79–1.43]	0.679
≤14%	540 (23.4)	221 (18.8)	0.90 [0.63–1.28]	0.562
Unknown	34 (1.5)	14 (1.2)	—	—
Geography				
Location^a^				
New England	175 (7.6)	118 (10.0)	Reference	
East South Central	206 (8.9)	98 (8.3)	0.63 [0.43–0.93]	**0.019**
West North Central	241 (10.4)	83 (7.0)	0.52 [0.35–0.76]	**0.001**
All others	1689 (73.1)	879 (74.6)	—	—

Note

Numbers might not sum to 100.0% due to rounding.

Abbreviations: AS, adjuvant surgery; CI, confidence interval; HR, hazard ratio.

—: Not calculated due to sample size.

^a^ Only includes regions with a statistically significant correlation.

Bold values denied statistical significance.

### 3.3 Factors associated with survival

**FIGURE 2:**
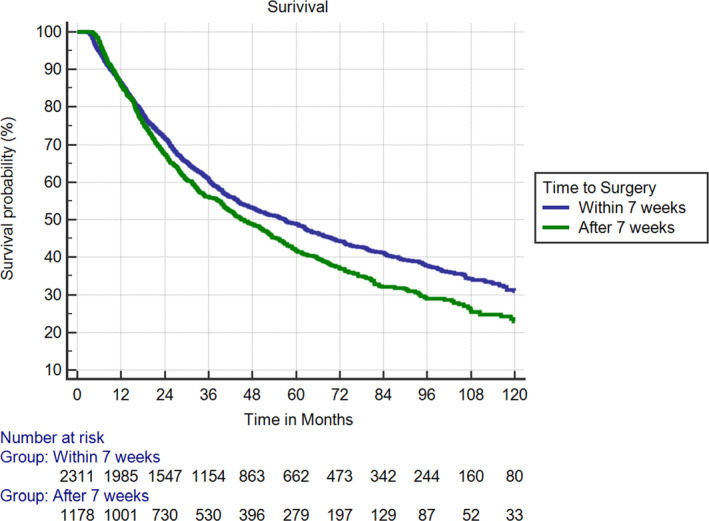
Kaplan–Meier survival curves for patients with surgery <7 weeks and >7 weeks. HR 1.18 [1.07–1.30]; *p* < 0.001. HR, hazard ratio

The median follow‐up time and OS for all patients were 57 months (IQR 42–99 months) and 51.9 months (95% CI 47.9–56.3 months), respectively. Median OS for the surgery ≤7 weeks group was 56.9 months compared with 45.6 months among the surgery >7 weeks group (HR 1.18 [1.07–1.30]; *p* < 0.001) (Figure 2). On MVA, older patients (HR 1.03 [1.02–1.03]; *p* < 0.001) and patients with increased burden of medical comorbidities denoted by a CDS of 2 (HR 1.28 [1.06–1.53]; *p* = 0.008) had statistically significantly worse survival. With regards to socioeconomic factors, female gender (HR 0.84 [0.76–0.93]; *p* < 0.001) and African American ethnicity (HR 0.82 [0.68–1.0]; *p* = 0.040) were significantly associated with improved OS. However, those living in an urban area adjacent to a metropolitan city with a population of 20,000–250,000 had a correlation with decreased OS (HR 1.26 [1.02–1.57]; *p* = 0.035). Education level and income had no association with OS. In terms of radiation technique, there were 510 and 2979 patients treated with intensity‐modulated radiation therapy (IMRT) and 3D‐conformal radiation therapy, respectively. There was no survival difference (HR=1.023 [0.893–1.171]) for IMRT, no >7 weeks (OR=1.04 [0.837–1.29]) for IMRT, or lobectomy >66 days for surgery (OR =1.05 [0.817–1.354]) for IMRT.

**FIGURE 3:**
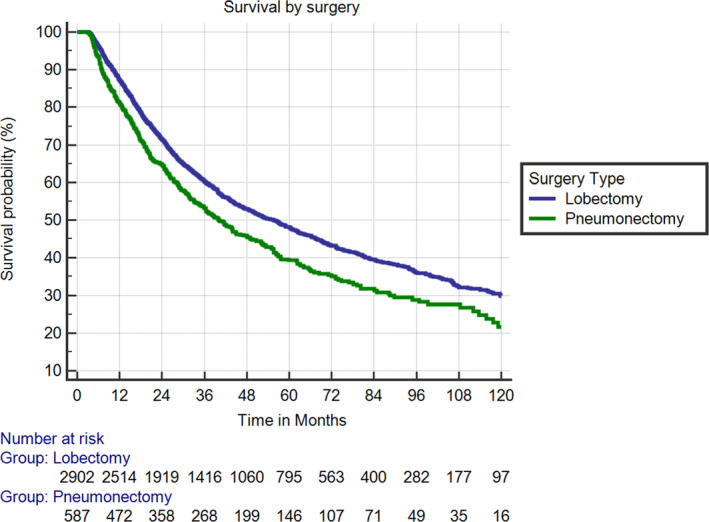
Kaplan–Meier survival curves for patients with lobectomy versus pneumonectomy. HR 1.33 [1.16–1.51]; *p* < 0.001. HR, hazard ratio

In general, a higher burden of the clinical disease led to inferior OS. On MVA, T4 disease (HR 1.24 [1.03–1.50]; *p* = 0.024), N2 disease (HR 1.29 [1.14–1.45]; *p* < 0.001), and N3 disease (HR 1.43 [1.08–1.90]; *p* = 0.014) were associated with decreased survival. Having surgery >7 weeks (HR 1.15 [1.04–1.27]; *p* = 0.008) and a pneumonectomy versus lobectomy (HR 1.33 [1.16–1.51]; *p* < 0.001) also had a significant association with decreased OS (Figure 3). In terms of histology, squamous cell correlated with a slight OS improvement in comparison to adenocarcinoma (HR 0.89 [0.80–0.98]; *p* = 0.023), driven primarily by pneumonectomy patients (Table 2). Radiation dose did not independently predict for OS. The median OS for patients treated with <50.4, 50.4–60, and >60 Gy were 53.9, 48.9, and 47.1 months, respectively (*p* = 0.31). Of the 2902 patients who underwent a lobectomy, their mortality rate was 5.2% when surgery occurred ≤66 days and 8.1% with surgery >66 days following NCRT.

### 3.4 Propensity score analysis

We conducted a PS‐matched analysis (1:1) of surgery ≤7 weeks (*N* = 772) to surgery >7 weeks (*N* = 772), with well‐balanced patient baseline characteristics between groups. Within this analysis, patients with surgery >7 weeks after NCRT had decreased OS (HR 1.16 [1.05–1.29]; *p* = 0.004) (Table 3). When we performed multivariable cox regression for survival with time to surgery as a continuous variable in days, we also found a significant correlation with decreased OS (HR 1.003 [1.001–1.006]; *p* = 0.006). This also remained statistically significant on PS‐matched analyses (HR 1.004 [1.001–1.006]; *p* = 0.004) (Table 4).^17^

**TABLE 3:** Multivariate analysis of treatment and disease characteristics associated with survival

	Multivariate	
Characteristic	HR [95% CI]	*p* value
Age	1.03 [1.02–1.03]	**<0.001**
Female gender	0.84 [0.76–0.93]	**<0.001**
Charlson–Deyo score		
0	Reference	
1	1.09 [0.97–1.21]	0.142
2	1.28 [1.06–1.53]	**0.008**
3	1.46 [0.96–2.23]	**0.077**
T stage		
1	Reference	
2	1.02 [0.87–1.19]	0.826
3	1.06 [0.90–1.25]	0.479
4	1.24 [1.03–1.50]	**0.024**
N stage		
0	Reference	
1	1.06 [0.90–1.24]	0.495
2	1.29 [1.14–1.45]	**<0.001**
3	1.43 [1.08–1.90] –	**0.014**
Histology squamous cell	0.89 [0.80–0.98]	**0.023**
Disease laterality		
Unknown	Reference	
Right	1.16 [0.83–1.63]	0.386
Left	1.23 [0.88–1.72]	0.230
Midline	1.23 [0.29–5.20]	0.775
Adjuvant surgery >7 weeks	1.15 [1.04–1.27]	**0.008**
Radiation dose (Gy)		
<50.4	Reference	
50.4–60	1.04 [0.91–1.18]	0.570
>60	1.05 [0.91–1.22]	0.475
Surgery		
Lobectomy	Reference	
Pneumonectomy	1.33 [1.16–1.51]	**<0.001**
Ethnicity		
White	Reference	
Black	0.82 [0.68–1.0]	**0.040**
Other	1.19 [0.88–1.61]	0.267
Facility type		
Community	Reference	
Comprehensive	1.06 [0.88–1.28]	0.535
Academic	1.04 [0.86–1.27]	0.670
Integrated	1.21 [0.96–1.51]	0.101
Other		
Income	NS in all groups	
No high school diploma	NS in all groups	
Population^a^		
Metro ≥1,000,000	Reference	
Urban ≥20,000–250,000	1.26 [1.02–1.57]	**0.035**
	Multivariate analysis with propensity score matching of survival	

Treatment	Overall survival Adjusted HR [95% CI]	*p* value
Sx ≤ 7 weeks	Reference	
Sx > 7 weeks	1.16 [1.05–1.29]	**0.004**

Abbreviations: CI, confidence interval; HR, hazard ratio; NS, not significant.

^a^ Only includes populations with a statistically significant correlation.

Bold values denied statistical significance.

**TABLE 4:** Multivariate and propensity score matched analysis of survival with time as a continuous variable

	Overall survival	
Treatment	Adjusted HR [95% CI]	*p* value
Multivariate	1.00 [1.001–1.006]	**0.006**
Propensity score matched	1.004 [1.001–1.0006]	**0.004**

Abbreviations: CI, confidence interval; HR, hazard ratio.

Bold values denied statistical significance.

### 3.5 Correlates of surgery >66 days in lobectomy patients

Of the 3489 patients with LA‐NSCLC patients treated with TMT, 2902 had a lobectomy for their surgical procedure. Of those, 1470 (50.7%) patients had surgery ≤66 days and 1432 (49.3%) >66 days. Both groups had a median age of 61 years. Baseline characteristics were similar between both groups (Table 5). Similar to those treated with surgery ≤7 weeks compared with >7 weeks, patients with lobectomy <66 days were more likely to have received a higher radiation dose compared to lobectomy >66 days. Specifically, 20.8% of patients with surgery >66 days were treated with a dose between 50.4 and 60 Gy versus 15.6% in surgery ≤66 days (*p* < 0.001). Furthermore, 20.8% of patients with surgery >66 days received a radiation dose >60 Gy versus 8.6% in the surgery ≤66 days (*p* < 0.001). Having treatment with higher radiation dose 50.4–60 Gy (HR 1.62 [1.22–2.15]; *p* < 0.001) and >60 Gy (HR 3.64 [2.78–4.76]; *p* < 0.001) compared to <50.4 Gy both predicted for lobectomy >66 days.

**TABLE 5:** Predictors of surgery (Sx) > 66 weeks in lobectomy patients

	Treatment group		Association with Sx > 66 days	
			Binomial regression	
Characteristic	Sx ≤ 66 days (*N* = 1470)	Sx > 66 days (*N* = 1432)	HR [95% CI]	*p*
Age (continuous)	61 (24–86)	61 (33–88)	1.00 [0.99–1.02]	0.668
Gender				
Male	801 (54.5)	800 (55.9)	Reference	
Female	669 (45.5)	632 (44.1)	1.24 [0.99–1.56]	0.057
Charlson–Deyo score				
0	961 (65.4)	908 (63.4)	Reference	
1	401 (27.3)	406 (28.4)	1.29 [1.01–1.65]	**0.039**
2	90 (6.1)	105 (7.3)	1.23 [0.80–1.88]	0.353
3	18 (1.2)	13 (0.9)	1.02 [0.36–2.86]	0.969
T stage				
T1	215 (14.6)	212 (14.8)	Reference	
T2	479 (32.6)	484 (33.8)	1.09 [0.77–1.53]	0.626
T3	495 (33.7)	479 (33.4)	1.30 [0.90–1.87]	0.157
T4	185 (12.6)	192 (13.4)	1.34 [0.88–2.04]	0.173
Unknown	96 (6.5)	65 (4.5)	—	—
N stage				
N0	478 (32.5)	443 (30.9)	Reference	
N1	177 (12.0)	173 (12.1)	1.16 [0.81–1.65]	0.427
N2	688 (46.8)	696 (48.6)	1.05 [0.78–1.42]	0.757
N3	28 (1.9)	37 (2.6)	1.26 [0.62–2.56]	0.517
Unknown	99 (6.7)	83 (5.8)	—	—
Histology				
Adenocarcinoma	786 (53.5)	746 (52.1)	Reference	
Squamous cell	684 (46.5)	686 (47.9)	1.11 [0.88–1.40]	0.372
Laterality				
Unknown	17 (1.2)	11 (0.8)	Reference	
Right	916 (62.3)	876 (61.2)	2.62 [0.58–11.92]	0.212
Left	534 (36.3)	544 (38.0)	2.61 [0.57–11.90]	0.215
Midline	3 (0.2)	1 (0.0)	6.99 [0.43–114.84]	0.174
Dose (Gy)				
<50.4	1113 (75.7)	836 (58.4)	Reference	
50.4 to ≤60	230 (15.6)	298 (20.8)	1.62 [1.22–2.15]	**<0.001**
>60	127 (8.6)	298 (20.8)	3.64 [2.78–4.76]	**<0.001**
Ethnicity				
White	1301 (88.5)	1258 (87.8)	Reference	
Black	120 (8.2)	148 (10.3)	1.35 [0.93–1.96]	0.115
Other	49 (3.3)	26 (1.8)	0.59 [0.26–1.36]	0.217
Facility type				
Community	123 (8.4)	101 (7.1)	Reference	
Comprehensive	663 (45.1)	653 (45.6)	0.65 [0.44–0.96]	**0.031**
Academic	486 (33.1)	495 (34.6)	0.85 [0.56–1.27]	0.418
Integrated	182 (12.4)	172 (12.0)	0.72 [0.44–1.18]	0.191
Unknown	16 (1.0)	11 (0.8)	—	0.998
Income				
<$30,000	226 (15.4)	273 (19.1)	Reference	
$30,000–$34,999	362 (24.6)	337 (23.5)	0.93 [0.65–1.32]	0.681
$35,000–$45,999	408 (27.8)	386 (27.0)	0.85 [0.58–1.27]	0.435
$46,000+	454 (30.9)	418 (29.2)	0.72 [0.45–1.17]	0.185
Unknown	20 (1.4)	18 (1.3)	—	—
No high school diploma				
≥29%	219 (14.9)	206 (14.4)	Reference	
≥20%–28.9%	374 (25.4)	415 (29.0)	1.07 [0.74–1.53]	0.730
≥14%–19.9%	518 (35.2)	502 (35.1)	1.15 [0.76–1.72]	0.508
≤14%	339 (23.1)	292 (20.4)	1.13 [0.69–1.85]	0.634
Unknown	20 (1.4)	17 (1.2)	—	—
Geography				
Location^a^				
New England	99 (6.7)	144 (10.0)	Reference	
Pacific	131 (8.9)	100 (7.0)	1.78 [1.01–3.12]	**0.044**
All others	1240 (84.4)	1188 (83.0)	—	—
Population^b^				
Metro >1,000,000	704 (47.9)	672 (46.9)	Reference	
Urban ≥20,000–250,000	72 (4.9)	81 (5.7)	1.27 [0.77–2.12]	0.349
Urban ≥2500–19,999	93 (6.3)	78 (5.4)	2.08 [1.14–3.79]	**0.017**
All others	601 (40.9)	601 (42.0)	—	—

Abbreviations: CI, confidence interval; HR, hazard ratio.

—: Not calculated due to sample size.

^a^ Only includes regions with a statistically significant correlation.

^b^ Only includes populations with a statistically significant correlation.

Bold values denied statistical significance.

### 3.6 Factors associated with postoperative mortality in lobectomy patients

Among all lobectomy patients, age (HR 1.05 [1.04–1.08]; *p* < 0.001) and CDS of 2 (HR 1.80 [1.02–3.17]; *p* = 0.042) compared to CDS of 0 correlated with increased postoperative mortality. Most tumor characteristics such as primary tumor stage, nodal stage, and tumor location did not have any statistically significant correlation with postoperative mortality. However, patients with N2 (HR 1.75 [1.08–2.84]; *p* = 0.024) disease versus N0 had increased postoperative mortality. Additionally, in lobectomy patients, SCC histology (HR 1.83 [1.25–2.68]; *p* = 0.002) was associated with worse survival.

For 30 day mortality, age (HR 1.05 [1.02–1.08]; *p* < 0.001) and pneumonectomy (HR 3.19 [1.92–5.31]; *p* < 0.001) but no other variables had statistically significant correlations with 30 day mortality on MVA. Treatment facility type was significantly associated with 90 day postoperative mortality; specifically comprehensive (HR 2.39 [1.04–5.47]; *p* = 0.039), integrated (HR 3.0 [1.18–7.56]; *p* = 0.021), and other non‐academic centers (HR 24.61 [3.62–167.18]; *p* = 0.001) had increased 90‐day mortality compared to academic centers. Finally, patients with surgery >66 days tended to have higher 90‐day postoperative mortality rates (HR 1.59 [1.02–2.50]; *p* = 0.040), but not 30‐day postoperative mortality rates (HR 1.02 [0.55–1.89]; *p* = 0.95) (Table 6). Radiation dose, however, did not independently predict for 90 day postoperative mortality.

**TABLE 6:** Multivariate analysis of lobectomy patients and disease characteristics associated with 90‐day postoperative mortality

Characteristic	HR [95% CI]	*p* value
Age	1.05 [1.04–1.08]	**<0.001**
Female gender	0.73 [0.50–1.06]	0.100
Charlson–Deyo score		
0	Reference	
1	0.92 [0.61–1.37]	0.676
2	1.80 [1.02–3.17]	**0.042**
3	1.41 [0.31–6.45]	0.658
T stage		
1	Reference	0.484
2	1.23 [0.69–2.22]	0.453
3	1.27 [0.68–2.38]	0.170
4	1.64 [0.81–3.34]	
N stage		
0	Reference	
1	0.75 [0.38–1.45]	0.388
2	1.75 [1.08–2.84]	**0.024**
3	2.26 [0.79–6.44]	0.128
Histology squamous cell	1.83 [1.25–2.68]	**0.002**
Disease laterality		
Unknown	Reference	
Right	0.54 [0.14–2.14]	0.383
Left	0.39 [0.10–1.56]	0.183
Surgery >66 days	1.60 [1.02–2.50]	**0.040**
Radiation dose (Gy)		
<50.4	Reference	
50.4–60	0.73 [0.44–1.19]	0.206
>60	0.60 [0.33–1.08]	0.091
Ethnicity		
White	Reference	
Black	0.47 [0.18–1.23]	0.125
Other	1.31 [0.43–4.01]	0.634
Facility type		
Community	Reference	
Comprehensive	2.39 [1.04–5.47]	**0.039**
Academic	1.90 [0.80–4.52]	0.148
Integrated	3.0 [1.18–7.56]	**0.021**
Other	24.61 [3.62–167.18]	**0.001**
Income	NS in all groups	
No high school diploma	NS in all groups	
Population		
Metro >1,000,000	Reference	
Urban ≥20.000	2.32 [1.08–5.01]	**0.032**
Urban ≥2,500–19,999	2.13 [1.00–4.51]	0.050
All others	NS	

Abbreviations: CI, confidence interval; HR, hazard ratio; NS, not significant.

Bold values denied statistical significance

## 4 DISCUSSION

In our analyses, we found that a lengthened TI of >7 weeks from NCRT to surgery significantly correlates with inferior OS for LA‐NSCLC. Patients undergoing lobectomy ≤66 days versus >66 days after NCRT have greater OS, though no differences exist in pneumonectomy patients. Among PS‐matched patients, the detriment to OS for surgery >7 weeks after NCRT persisted. On MVA, time as a continuous variable had a significant association with OS, which remained on PS matching. The mortality rate was higher in patients treated >66 days after the completion of NCRT, and this remained statistically significant on univariate analysis, while remaining independently associated with MVA. Among all 587 patients treated with pneumonectomy, OS was not influenced by TI. The results from our analyses underscore the necessity of careful pretreatment selection of patients eligible for TMT to maximize the efficacy and safety of this approach.

Per National Comprehensive Cancer Network, a multidisciplinary thoracic oncology team should determine the resectability of potential TMT candidates before beginning any treatment. However, in clinical practice nationally, not all patients are discussed in a multidisciplinary setting, with a recent cross‐sectional survey reporting that only approximately 55% of stage III NSCLC patients presented at live or virtual tumor boards.^18^ In Intergroup 0139, Albain et al.^5^ found significant complications from TMT, specifically a 26% postoperative mortality rate in pneumonectomy patients. Several prospective trials called for patients to undergo surgery 3–5 weeks ^1^, ^2^, ^3^, ^5^ after completing NCRT, whereas others allowed up to 8 weeks,^6^ raising the question of whether an optimal TI to surgery after NCRT exists, and whether or not timing affects OS and postoperative mortality.

In terms of neoadjuvant RT dose, wide individual and institutional variability exists,^19^ with some centers using 45 Gy, previously established in phase II and III cooperative group trials,^1^, ^2^, ^3^, ^5^ while others standardly employ dose escalation in the neoadjuvant setting.^4^, ^20^ As such, NCRT dose and TI to surgery are often determined by the physician and/or institutional preference, and they are not currently based on high‐level evidence.

Our finding that TI of >7 weeks between the completion of NCRT and having surgery results in worse OS is 1 week more than the 6‐week threshold established by Gao et al.^7^ They reported significant drops in OS in patients with surgery >6 and ≤9 weeks and >9 and ≤12 weeks after NCRT. However, differences exist between our analyses, most notably the exclusion of T4 N0‐1 and stage IIIB disease. In addition, they limited the radiation dose to a maximum of 60 Gy. However, several cooperative group prospective trials, including SWOG 0220^3^ and SWOG 9416,^1^ included T4N0‐1 tumors, and SWOG 8805^2^ also included select N3 disease. The exclusion of these patients plus our use of a more recent database may account for the difference in findings. Another analysis by Rice et al.^8^ found that patients who received any neoadjuvant treatment followed with surgery had significantly decreased survival when comparing surgery <77 days to surgery >114 days. They included patients treated with any neoadjuvant treatment, whether it was chemotherapy, RT, or concurrent chemoradiation. However, several studies demonstrate varying degrees of treatment‐related toxicity depending on the neoadjuvant therapy regimen, with clear differences expected between systemic therapy alone and concurrent chemoradiation.^21^, ^22^, ^23^ In general, concurrent chemoradiation is thought to result in worse treatment‐related toxicity such as radiation pneumonitis than chemotherapy alone, which may be further exacerbated with the addition of surgery with TMT.^24^ In RTOG 9410,^25^ the addition of RT concurrently to chemotherapy synergistically worsened grade ≥3 esophagitis (4% vs. 22%). We, therefore, specifically examined the patient population receiving NCRT before surgery.

While prior studies have not assessed for or found a significant correlation with higher radiation dose and increased TI to surgery or increased 90‐day postoperative mortality in lobectomy patients who had delayed surgery, our analysis is the first on this topic to have also conducted a propensity‐matched‐pair analysis, which reduces bias created by confounding variables, ultimately strengthening our results. We identified radiation dose as a predictor of prolonged TI to surgery; >7 weeks to surgery and >66 days to lobectomy. Higher NCRT dose between 50.4 and 60 Gy and dose >60 Gy compared to dose <50.4 Gy significantly correlated with surgery >7 weeks on MVA. Having surgery >7 weeks and a pneumonectomy versus lobectomy both had a statistically significant association with decreased OS. This parallels the findings from Intergroup 0139,^5^ where lobectomy patients had increased median survival and 5‐year survival compared to bimodality patients.

In patients treated with lobectomy, we also found that treatment with higher neoadjuvant RT dose of both 50.4 to 60 Gy and >60 Gy compared to <50.4 Gy predicted for lobectomy >66 days. Furthermore, patients with surgery >66 days tended to have higher 90‐day postoperative mortality rates. As such, a dose of 44–50.4 Gy may minimize the risk of the development of radiation‐induced pulmonary toxicity, which as an early surrogate can be signified by a decline in pulmonary function tests and impaired the diffusion capacity of lung for carbon dioxide. Lower doses may also decrease surgical complications and potentially allow for quicker recovery of pulmonary function, resulting in surgery within the optimal time point of <7 weeks. NCRT impairs diffusion capacity of lung for carbon monoxide^26^ and these effects seem largely to last 4–6 weeks for event resolution.^27^ Higher radiation doses may increase the severity of these effects, prolong their time to resolution, and increase patient recovery time, which ultimately increases TI to surgery. However, it is difficult to specifically determine what contributed to higher radiation doses being associated with surgery >7 weeks and lobectomy >66 days due to the NCDB’s omission of variables specific for treatment complications and/or morbidity. Notably, however, radiation dose did not independently predict of OS or postoperative mortality in this analysis. While radiation dose escalation in the neoadjuvant setting can improve the rate of neoadjuvant nodal clearance,^4^, ^11^ and while such higher radiation doses have led to notable survival times for trimodality patients at experienced centers, we recommend dose escalation above 50.4 Gy when treating on a clinical trial or at high volume thoracic centers with surgeons experienced with operating following higher neoadjuvant RT doses, as therapy at high volume centers correlates with superior OS for NSCLC patients treated with chemoradiation^28^ and for patients with other thoracic malignancies treated with TMT.^29^

Several limitations to our analysis exist. Due to the lack of information provided in the database and coding, we included a small number of early‐stage lung cancer patients (3.5%) and patients treated with doses RT higher than typically employed for TMT. However, approximately 90% of the patients included in our analyses were stage III patients. Furthermore, a subset multivariable regression analysis for true stage III patients demonstrated that the independent correlates of prolonged TIs and survival were unchanged. And as such, given that locally advanced NSCLC is quite a heterogenous population, there is a value in reporting on pragmatic and real‐world experiences of trimodality in the minority of patients who are N1 and receive that treatment approach, although the numbers of patients in the non‐stage III cohorts are limited.

Furthermore, the NCDB does not report information regarding specific chemotherapy treatments or dose, and so further analyses on effects of specific chemotherapy agents and dosing were not possible. However, treatment‐induced lung injury can be impacted by the type of cytotoxic systemic treatment administered with radiation therapy,^30^ as well as by targeted therapies such as tyrosine kinase inhibitors that may be delivered with chemotherapy and RT.^31^ High radiation dose combined with concurrent chemotherapies and targeted agents may increase inflammation and pulmonary injury that increases TI to surgery, although the severity and duration of such effects may vary based on the specific agent used.^32^ While survival information is robust in the NCDB, the database would not allow for the identification of a potential new site of disease identified between neoadjuvant chemoradiotherapy and surgery on preoperative restaging, which may have affected the decision of surgery in a small minority of patients.

In addition, this database does not provide reasons why a particular radiation dose was chosen. In other retrospective reviews, patients treated with curative intent full‐dose chemoradiation were referred for early salvage surgery when faced with persistent non‐nodal disease.^33^ Therefore, patients receiving doses ≥60 Gy may have originally been considered for definitive treatment, and these patients may have had the residual disease or very early recurrence following concurrent chemoradiation. We attempted to mitigate the latter population from being included in this analysis by excluding patients who underwent surgery >4 months following the completion of RT. Moreover, patients treated with lower dose may have originally been intended to have TMT and potentially better responses to induction therapy.

Additionally, we are unable to determine whether patients were restaged after NCRT with a mediastinoscopy to confirm persistent N2 or N3 disease. In addition, the methods used for determining mediastinal stage are not available, and patients originally clinically staged with positive mediastinal lymph node disease may never have had biopsy‐confirmed tissue. Furthermore, although CDS provides general baseline information about a patient's overall health, specifics regarding performance status, pulmonary function, and cardiac disease, which may affect radiation‐induced lung injury, risk of perioperative mortality, and OS, are lacking. The database does not provide reasons why surgery may have been delayed following NCRT. Finally, the option of planned surgery is most optimally considered during an initial pre‐treatment multidisciplinary tumor board discussion, as opposed to after the completion of CRT. NCDB, however, does not provide access to details of initial pre‐treatment tumor board decisions.

Despite these limitations, this is the largest analysis dedicated to assessing timing and dosing of NCRT in TMT patients, and our results remained consistent and robust with several statistical analyses such as multivariate stratification and PS matching, which support TI to surgery <7 weeks. An increased TI of >7 weeks from NCRT to surgery correlates with inferior OS for LA‐NSCLC patients and those treated with lobectomy >66 days have decreased OS. However, due to the retrospective nature of the NCDB database, a definitive recommendation with this TI should be interpreted with caution. In the future, genomic and radiomic predictors and circulating tumor products^34^ may aid in predicting locoregional and distant control in LA‐NSCLC, allowing clinicians to better tailor individual patient treatment. Until then, clinician judgment should be used to select the most appropriate candidates for TMT and to determine the optimal radiation dose and time to surgery. Future phase III trials are necessary to validate these results and provide further information with regards to NCRT dose and optimal TI to surgery in TMT patients.

## CRediT Statement

**James E. Han:** conceptualization, formal analysis, writing – original draft, visualization. **Shaakir Hasan:** conceptualization, methodology, software, validation, formal analysis, supervision, writing – review and editing. **J. Isabelle Choi:** writing – review and editing. **H. Robert Press:** writing – review and editing. **Charles B. Simone:** supervision, conceptualization, writing – review and editing.

## CONFLICTS OF INTEREST

All the authors do not have any conflicts of interest related to this manuscript.

## DISCLOSURES

CBS reports a Varian Medical Systems honorarium outside of the submitted work.

## Data Availability

The data that support the findings of this study are available from the corresponding author upon reasonable request.
